# Moderation analysis in two-instance repeated measures designs: Probing methods and multiple moderator models

**DOI:** 10.3758/s13428-018-1088-6

**Published:** 2018-10-10

**Authors:** Amanda Kay Montoya

**Affiliations:** 1 Ohio State University, Columbus, OH USA; 2 Department of Psychology, University of California–Los Angeles, 1285 Franz Hall, Los Angeles, CA 90095 USA

**Keywords:** Linear regression, Moderation, Repeated measures, Interaction, Probing, Johnson-Neyman

## Abstract

Moderation hypotheses appear in every area of psychological science, but the methods for testing and probing moderation in two-instance repeated measures designs are incomplete. This article begins with a short overview of testing and probing interactions in between-participant designs. Next I review the methods outlined in Judd, McClelland, and Smith (*Psychological Methods* 1; 366–378, 1996) and Judd, Kenny, and McClelland (*Psychological Methods* 6; 115–134, 2001) for estimating and conducting inference on an interaction between a repeated measures factor and a single between-participant moderator using linear regression. I extend these methods in two ways: First, the article shows how to probe interactions in a two-instance repeated measures design using both the pick-a-point approach and the Johnson–Neyman procedure. Second, I extend the models described by Judd et al. (1996) to multiple-moderator models, including additive and multiplicative moderation. Worked examples with a published dataset are included, to demonstrate the methods described throughout the article. Additionally, I demonstrate how to use Mplus and MEMORE (Mediation and Moderation for Repeated Measures; available at http://akmontoya.com), an easy-to-use tool available for SPSS and SAS, to estimate and probe interactions when the focal predictor is a within-participant factor, reducing the computational burden for researchers. I describe some alternative methods of analysis, including structural equation models and multilevel models. The conclusion touches on some extensions of the methods described in the article and potentially fruitful areas of further research.

Across areas of experimental psychology and many other scientific fields, researchers are interested in questions that address the boundaries and contingencies of certain effects they observe. Do women feel more comfortable around men after learning their sexual orientation, or does it depend on whether the man is hetero- or homosexual (Russell, Ickes, & Ta, 2018)? Does fear-based advertisement always work, or will thinking about God make these methods less effective (Wu & Cutright, 2018)? Are all veterans equally likely to experience post-service stress, or will certain psychological characteristics impact the risk of stress (Mobbs & Bonanno, 2018)? These are all questions of *moderation* or *interaction*. Though some differentiate between these two terms, I will use them interchangeably (see VanderWeele, 2009, for a discussion of the differences from a causal modeling perspective). Statistical moderation analysis is used to test whether the relationship between a *focal predictor*, *X*, and an *outcome variable*, *Y*, depends on some *moderator*, *W*. For example, Kraus and Callaghan (2016) found that higher-class individuals were more likely to help than lower-class individuals in public contexts, but the opposite was true when the context was private, where lower-class individuals helped more than higher-class individuals. Here, the relationship between class (*X*) and helping (*Y*) depended on context (*W*). Learning has been shown to improve when adjunct questions are included in a text, but Roelle, Rahimkhani-Sagvand, and Berthold (2017) found that when reading texts with adjunct questions, receiving immediate feedback (*X*) had a detrimental effect on learning (*Y*) for students who felt that answering the questions was highly demanding (*W*). So, how is social class related to helping? Does immediate feedback lead to worse learning outcomes? It depends. Moderation analysis is a statistical method for testing whether these relationships depend on certain proposed variables (i.e., moderators).

In moderation analysis we test whether the relationship between the focal predictor (*X*) and the outcome (*Y*) depends on the moderator (*W*). If the analysis suggests that the answer is “Yes,” the next natural question is “How?” An interaction can look many different ways, and the practical implications of significant interactions often depend on how the relationship between *X* and *Y* changes across the range of *W*. For example, the relationship between *X* and *Y* can increase as *W* increases, or the relationship between *X* and *Y* can decrease as *W* increases. A hypothesis test of moderation would say the same thing for each of these patterns: “Yes, there is significant moderation.” Because each pattern tells a different story, a follow-up analysis is required to interpret these effects.

One way to understand moderation is by estimating and probing *conditional effects*. A conditional effect is the effect of one variable on another, conditioned on a third. In analysis of variance, these are called *simple effects*. In moderation analysis, researchers are typically interested in the conditional effect of *X* on *Y* at different values of *W.* This helps researchers better understand how the relationship between *X* and *Y* changes as *W* changes. Probing an interaction gives us information about the nature of this changing relationship. For example, imagine you are researching how after-school science experience (*X*; e.g., in a science club) predicts performance in science classes (*Y*), and whether the effect differs by gender (*W*). If you find an interaction between experience and gender, you know that the effect of after-school science experience is different for males than for females. The next questions you might ask are “Does after-school experience help boys but not girls?,” “Does it help girls but not boys?,” and “If after-school experience helps both boys and girls, is the effect stronger for one gender?” Probing the interaction can help answer these questions. This is done by estimating the effect of *X* on *Y* at a certain point (or points) along the moderator, and testing whether this effect is significantly different from zero. Directional tests can also be used to understand not just whether an effect is different from zero, but also whether it is positive or negative. Information about where effects are positive, indistinguishable from zero, and negative helps you understand the pattern of effects across the moderator.

## Rationale and summary

Moderation hypotheses can be investigated using a variety of experimental designs; however, the methods for conducting moderation analysis are not equally developed in all designs. Here, I focus on two designs: between-participant designs (e.g., participants are randomly assigned to condition; participants are observed once on each outcome of interest) and two-instance repeated measures designs (e.g., participants experience both conditions or are measured twice over time; participants are observed twice on each outcome of interest). Both designs are very common in psychology and other behavioral sciences. The defining difference between the two designs is that each participant is observed on each outcome only once in between-participant designs. In contrast, repeated measures designs observe each participant multiple times (e.g., over time, in multiple situations).

Methods for testing and probing interactions in between-participant designs have been established, and it has become typical for graduate students to learn how to conduct these analyses in an introductory regression course. Easy-to-use tools have been developed to help researchers conduct moderation analyses and probe interactions in between-participant designs (e.g., Hayes, 2018; Preacher, Curran, & Bauer, 2006). However, less is known about how to test moderation effects when either the moderator or the focal predictor is a within-participant factor. Judd and colleagues (Judd, Kenny, & McClelland, 2001; Judd, McClelland, & Smith, 1996) have provided the only treatments of this topic in a linear regression framework. Their two articles discuss moderation of the effect of a repeated measures treatment on some outcome by a variable that is measured once and assumed to be constant over instances (I call this a *between-participant variable*).

In this article, I begin by providing a short overview of testing and probing interactions in between-participant designs. Then I review the methods outlined by Judd et al. (2001, 1996) for estimating and conducting inference on interactions between repeated measures factors and between-participant variables using a linear regression approach. The primary purpose of this article is to extend the methods proposed by Judd et al. (2001, 1996) in two ways. First, I will explain how to probe interactions in a two-instance repeated measures design, a topic that has not yet been discussed in the methodology literature. Second, I extend the Judd et al. (1996) method to multiple moderator models, including additive and multiplicative moderation. Using published data, I provide a one moderator example and a two moderator example, both with repeated measures factors as focal predictors. Throughout the article, I will demonstrate how to use MEMORE (Mediation and Moderation for Repeated Measures; available at https://www.akmontoya.com), an easy-to-use tool available for SPSS and SAS, to estimate and probe interaction effects in which the focal predictor is a within-participant factor, reducing the computational burden for researchers. I also include Mplus code for estimating and probing these types of models. I conclude with alternatives and extensions of the proposed models as well as future directions, which include more than two instances, alternative models of change, and moderated mediation in two-instance repeated measures designs.

## Advantages of repeated measures designs

Repeated measures designs often boast some statistical and conceptual advantages over between-participant designs. In particular, with a repeated measures design each person can act as their own control. For instance, imagine a study in which participants read two stories, one that was sad and one that was happy. For each story they rate how likely they would be to help the main character. Helping tendency can vary a lot from person to person, with some people willing to help just about anyone and others who are the opposite. Here, a repeated measures design would be very advantageous because participants’ helping response from the sad story can be compared to their helping response from the happy story. In a within-participant design, the statistical noise due to individual differences can be avoided, making the estimate of the effect of story valence (sad vs. happy) on helping much more precise. When it is feasible for participants to be observed in all potential conditions in the study (i.e., when participating in multiple conditions is possible without concerns of carryover effects), there is a distinct statistical power advantage in using a repeated measures design.

A particular advantage of a within-participant design is the improved ability to observe causal effects on an individual. Like in between-participant designs specific assumptions are still required. When researchers are interested in cause and effect, they are typically interested in how some treatment might change an individual person. In a between-participants design, treatments are assigned to different sets of individuals and if treatment is randomly assigned, then group differences reflect a causal effect of the treatment. Here, inferential statements are limited to what a person *would have been like* if they had been in the other treatment condition, and this causal effect cannot be directly observed on any given individual. In a within-participant design, the information about what each participant would have been like in each condition is known. However, we do not know what each person would have been like if we had observed them in each condition in a different order. To observe causal effects, we must assume there are no carryover or order effects from the first observation to the second (i.e., causal transience), and there must also be an assumption of temporal stability, which means that the observed measurements do not depend on *when* they are measured (Holland, 1986). One particular advantage of the design and analysis described in this article is that, if order is counterbalanced across individuals, then order can be tested as a between-subjects moderator and the causal transience assumption can be tested statistically. However, it is important to note that traditional null hypothesis testing procedures cannot provide support for the claim of causal transience, as this would involve accepting the null hypothesis; rather, these tests can be used to detect violations of this assumption. Testing procedures such as equivalence testing could potentially be used to support the assumption of causal transience. If treatment order is randomized and balanced, the assumption of temporal stability is not required in order for estimates of the average causal effect to be unbiased (Josephy, Vansteelandt, Vanderhasselt, & Loeys, 2015; Senn, 2002).

## Moderation in between-participant designs

Before discussing moderation in repeated measures designs, I review the principles of testing and probing interactions in between-participant designs, in order to connect methods from this design to those for repeated measures designs. More extensive introductions to moderation analysis in between-participant designs can be found in Hayes (2018), Jaccard and Turrisi (2003), Aiken and West (1991), and many other articles and books. In a between-participant design, each participant is measured once on all variables involved: the focal predictor, moderator, and outcome variable.

In a standard multiple linear regression, the relationship between *X* and *Y* is constant with respect to *W.* Researchers can test for moderation by allowing the relationship between *X* and *Y* to be a function of *W*, *f*(*W*_*i*_). In psychology, the form for *f*(*W*_*i*_) is typically a linear function of the moderator, *W* (e.g., *f*(*W*_*i*_) = *b*_1_ + *b*_3_*W*_*i*_).

1 $$ {\displaystyle \begin{array}{c}{Y}_i={b}_0+\left({b}_1+{b}_3{W}_i\right){X}_i+{b}_2{W}_i+{\epsilon}_i\\ {}={b}_0+{b}_1{X}_i+{b}_2{W}_i+{b}_3{X}_i{W}_i+{\epsilon}_i\end{array}} $$

In this model, the outcome variable for participant *i*, *Y*_*i*_, is a function of both participant *i*’s responses on focal predictor, *X*_*i*_, and their response on the moderator, *W*_*i*_. The error in estimating person *i*’s response on *Y*_*i*_ with this combination of *X*_*i*_ and *W*_*i*_ is represented by *ϵ*_*i*_. The coefficient *b*_0_ corresponds to the intercept (predicted value of *Y* when *X* and *W* are both zero). The relationship between *X* and *Y* is a linear function of *W*: *b*_1_ + *b*_3_*W*_*i*_. The coefficient *b*_2_ can be interpreted as the relationship between *W* and *Y* when *X* is zero. This equation can be estimated using any multiple linear regression program. When *b*_3_ is zero, the relationship between *X* and *Y* does not depend on *W* (i.e., *b*_1_ + 0*W* = *b*_1_). A test on ${\widehat{b}}_3$ is a test of moderation. (Hat notation denotes an estimate of a parameter).

The symmetry property of moderation states that if the relationship between the focal predictor and the outcome depends on the moderator, this also means that the relationship between the moderator and the outcome variable depends on the focal predictor. By manipulating Eq. 1, it is clear that *b*_3_ also measures the degree to which the relationship between *W* and *Y* depends on *X*.

$$ {Y}_i={b}_0+{b}_1{X}_i+\left({b}_2+{b}_3{X}_i\right){W}_i+{\epsilon}_i $$

If there is evidence of moderation, the researcher’s focus will shift toward the pattern of effects. The effect of one variable on another can depend on a third in many ways, and probing the interaction helps describe that pattern. The function *b*_1_ + *b*_3_*W*, which I will denote *θ*_*X* → *Y*_ (*W*), is the *conditional effect of X on Y*, which is a function of *W*. While probing an interaction, *θ*_*X* → *Y*_ (*W*) is estimated and inference is conducted at different values of *W*. The researcher can select values of *W* and use the estimate of the conditional effect and its standard error to test if the relationship between *X* and *Y* is significantly different from zero at that value of *W*. There are two primary methods of probing an interaction: the pick-a-point approach and the Johnson–Neyman procedure.

Probing analyses are typically most informative when a test of interaction is significant, though there may be justifiable reasons why a researcher would want to probe an interaction that is not significant. A hypothetical example might be if a strong existing literature supported the relationship between *X* and *Y* in one group (e.g., heterosexual couples), but little was known about that relationship in another other group (e.g., homosexual couples). If the couple’s sexual orientation were the moderator, it might be worth examining the relationship between *X* and *Y* in the heterosexual group to confirm that the previous results were replicated. A brief warning: Researchers who probe nonsignificant interactions often find themselves grappling with explaining seemingly contradictory results. For example, it may be that the relationship between *X* and *Y* is not significantly moderated by sexual orientation of the couple. However, when probed the analysis might show the relationship between *X* and *Y* is significantly different from zero for heterosexual couples, but not significantly different from zero for homosexual couples. It is important to remember that a *difference in significance does not imply significantly different*. One conditional effect may be significantly different from zero and other may not. This does not mean that these two conditional effects are significantly different from each other.

### Pick-a-point

The pick-a-point approach (i.e., simple-slopes analysis or spotlight analysis) is a method for probing an interaction by selecting a value of the moderator then estimating and conducting inference on the conditional effect of the focal predictor on the outcome at that specific value of *W*. This helps answer the question “Is there an effect of *X* on *Y* at *w*?,” where *w* is a specific point on *W*.

The ratio of the estimate ${\widehat{\theta}}_{X\to Y}(W)$ to its standard error can be compared to values from a *t*-distribution with *n* − *q* − 1 degrees of freedom, where *n* is the total number of participants and *q* is the number of predictors in the regression equation used to estimate the coefficients and standard errors. By comparing to the *t*-distribution a *p* value can be calculated, or the critical *t* value can be used to calculate a confidence interval*.*

The pick-a-point approach can be used for either dichotomous or continuous moderators. The choice of points to probe in the pick-a-point approach is very clear when the moderator is dichotomous. However, when the moderator is continuous the choice is often arbitrary. It has traditionally been recommended to select the mean of the moderator as well as the mean plus and minus one standard deviation (Aiken & West, 1991). However, these points may or may not be within the observed range of the data. Hayes (2018) recommends probing at percentiles (e.g., 16th, 50th, and 84th) to guarantee that the probed points are always within the observed range of the data. There are also instances in which certain points on the scale are particularly informative. For example, if the moderator is body mass index, then 18.5, 25, and 30 might be good points to probe as they indicate the boundaries between underweight, normal, overweight, and obese. For detailed discussions of the pick-a-point approach for between-participant designs, see Hayes and Matthes (2009), Aiken and West (1991), Hayes (2018), and Spiller, Fitzsimons, Lynch, and McClelland (2013).

### Johnson–Neyman procedure

The Johnson–Neyman procedure is another approach to probing interactions with continuous moderators (Johnson & Fay, 1950; Johnson & Neyman, 1936). This method removes the arbitrary choice of points along the moderator, and instead this method identifies important transition points (i.e., *boundaries of significance*) where the effect of the *X* on *Y* transitions from significant to nonsignificant, or vice versa. The procedure uses the same point estimate and standard error as the pick-a-point approach. However, rather than selecting a value of *W*, and calculating the associated *t*-statistic, the Johnson–Neyman procedure selects an *α* value and the associated critical *t* value, then solves for the values of *W* such that the conditional effect of *X* on *Y* is exactly significant at the preselected *α* value. This is done by setting the ratio of ${\widehat{\theta}}_{X\to Y}(W)$ to its standard error equal to the critical *t* and solving for *W*.

Solving for *W* involves finding the roots of a polynomial equation, and as such the Johnson–Neyman procedure is limited to continuous moderators (Hayes & Matthes, 2009). The solutions can be imaginary or outside of the range of the observed data. Methodologists do not recommend interpreting these solutions (Hayes & Matthes, 2009; Preacher et al., 2006; Spiller et al., 2013). By finding the transition points that lie within the observed data, this method allows the researcher to understand the patterns of significance across the entire range of the moderator, rather than at arbitrarily selected points.

## Moderation in two-instance repeated measures designs

Moderation analysis and probing have been widely described in between-participant designs, resulting in an increasing use of methods and new tools that made probing interactions easier (Hayes, 2013; Hayes & Matthes, 2009). Recent advances in probing have generalized these methods to multilevel modeling and latent curve analysis (Bauer & Curran, 2005; Preacher et al., 2006). However, the two-instance repeated measures design has been largely ignored when it comes to probing methods. Judd et al. (2001, 1996) described methods for estimating and testing whether there is an interaction, and these methods have been used across areas of psychology to investigate questions of moderation. For example, among students with math difficulties, those with higher working memory capacity benefited more from strategy training (pre- to posttest) than those with lower working memory capacity (Swanson, Lussier, & Orosco, 2015). In another example, dyads were assigned to complete a paired task, and partners were randomly assigned to high- or low-power roles. The dyad members in high-power positions were more likely to dehumanize the low-power participant than vice versa, particularly when the high-power individual offered fewer resources to the low-power individual (Gwinn, Judd, & Park, 2013).

In this section I review the methods described by Judd et al. (2001, 1996) for testing whether some between-participant variable moderates the effect of a repeated measures factor on an outcome. I refer to the repeated measures factor as either an “instance” or “condition,” with the understanding that what differentiates the repeated measurements does not need to be an experimental manipulation. For example, in the dataset used in this article, participants were measured at two time points: before and after treatment. The between-participant variable can be something that is randomly assigned (e.g., experimental condition), or it can be something that is observed but assumed to be constant across instances of repeated measurements (e.g., eye color). I’ve created a macro, available for SPSS and SAS, that both estimates the model required to test moderation and gives extensive output related to the probing methods described in this article. This tool, MEMORE, is meant to ease the computational burden of conducting a thorough moderation analysis in the two-instance repeated measures design. Though not described in this manuscript, MEMORE can also be used to estimate mediation models in two-instance repeated measures designs (for instructions and examples, see https://www.akmontoya.com and Montoya & Hayes, 2017).

### Running example

Lasselin, Kemani, Kanstrip, Olsson, Axelsson, Andreasson, and colleagues (2016) investigated whether baseline inflammation moderated the effectiveness of behavioral treatment for chronic pain. They were particularly interested in whether the treatment was less effective for individuals with higher baseline inflammation. Patients with chronic pain were recruited to the study, which involved 12 weekly sessions using one of two behavioral treatments for chronic pain: acceptance and commitment therapy (ACT) or applied relaxation (AR). Participants reported their pain on a 6-point scale before starting treatment and after completion of the sessions. Baseline inflammation was measured by taking assays of two pro-inflammatory markers (IL-6 and TNF-*α*). The concentrations of these markers were log transformed to improve linearity and combined using principal components analysis. The analyses in this article differ slightly from the analyses in Lasselin et al. (2016). A single outlier was dropped for all analyses in this article. All tests are conducted at *α* = .05. For the original analysis and other variables measured in the study, see Lasselin et al. (2016). The data for this example are available for download at https://www.akmontoya.com. All analyses are described as if the data are in wide form (each row of the dataset represents a participant) rather than long form (each row of the dataset represents a participant in a specific instance).

### Testing the interaction

An interaction means that the slope that describes the relationship between the focal predictor and the outcome depends on some other variable, a moderator. Judd et al. (2001, 1996) used this idea of varying slopes to outline the following method for testing interactions between a between-participant variable and a repeated measures treatment. This procedure begins with a model of the outcome variable *Y* predicted by *W*, the between-participant variable, where the regression weights for this model are allowed to vary by condition or instance (denoted using the subscript *j*):

$$ {Y}_{ij}={b}_{0j}+{b}_{1j}{W}_i+{\epsilon}_{ij} $$

*Y*_*ij*_ is the measure of the outcome *Y* for participant *i* in condition or instance *j*. The measurement of participant *i* on the between-participant variable is denoted *W*_*i*_. Notice that this measurement does not have a subscript *j* because it is not measured repeatedly, but rather assumed to be constant regardless of instance or condition. The intercept and regression weight for *W*_*i*_ are represented by *b*_0*j*_ and *b*_1*j*_, respectively. Note that if there are two instances, there are two *b*_0*j*_s: *b*_01_ and *b*_02_. Similarly, there would also be two *b*_1*j*_s. The intercept and slope are allowed to differ by condition. The *ϵ*_*ij*_s are the errors in estimation for participant *i* in condition *j* and are assumed to be normally distributed with mean zero, variance ${\sigma}_j^2$, correlation *ρ* for observations from the same participant, and correlation of 0 for observations from different participants.

In the case of two conditions there are two models, one for each outcome variable:

2 $$ {Y}_{i1}={b}_{01}+{b}_{11}{W}_i+{\epsilon}_{i1} $$

3 $$ {Y}_{i2}={b}_{02}+{b}_{12}{W}_i+{\epsilon}_{i2} $$

The coefficient *b*_11_ represents the relationship between *W* and *Y* in the first condition. The coefficient *b*_12_ represents the relationship between *W* and *Y* in the second condition. When *b*_11_ ≠ *b*_12_, the relationship between *W* and *Y* depends on the condition (i.e., there is an interaction between condition and *W*). To test a moderation hypothesis, we test whether *b*_11_ is equal to *b*_12_. By subtracting Eq. 2 from Eq. 3, the coefficient for *W* reflects the difference between *b*_11_ and *b*_12_.^1^

4 $$ {\displaystyle \begin{array}{c}{Y}_{i2}-{Y}_{i1}={b}_{02}-{b}_{01}+\left({b}_{12}-{b}_{11}\right){W}_i+\left({\epsilon}_{i2}-{\epsilon}_{i1}\right)\\ {}{Y}_{Di}={b}_0+{b}_1{W}_i+{\epsilon}_i\end{array}} $$

Regress the difference of the *Y* variables *Y*_*i*2_ − *Y*_*i*1_ = *Y*_*Di*_ onto *W*, and if *b*_1_ is significantly different from zero, we conclude that there is evidence for an interaction between *W* and condition. This implies that *b*_11_ and *b*_12_ are not equal, which means the relationship between *W* and *Y* depends on the condition. This matches the intuitive understanding of an interaction. Equation 4 is referred to as a *simple moderation model*, where “simple” refers to their being one moderator, *W* (similar to “simple regression” referring to one predictor). The symmetry property holds in this model: support for the claim that the relationship between condition and *Y* depends on *W* is the equivalent to saying the relationship between *W* and *Y* depends on condition.

#### Estimation with MEMORE

In Lasselin et al. (2016), the researchers were interested in whether the effect of behavioral treatment for chronic pain on pain intensity depended on baseline inflammation. MEMORE can be used to estimate this regression. One advantage of MEMORE is that the researcher does not need to create the difference score as an additional variable. Rather, after installing MEMORE, the researcher can input the two outcome variables using a MEMORE command line. If the variables for pre-treatment pain and post-treatment pain are stored as PrePain and PostPain, respectively, and the variable for pre-treatment inflammation is saved as inflame, then the MEMORE command for this analysis in SPSS would be
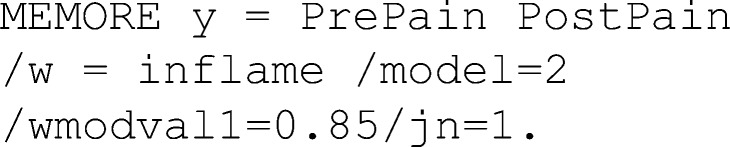


The command MEMORE calls the MEMORE macro. The instructions for installing the MEMORE macro can be found at https://www.akmontoya.com. The variables in the y list are used as the outcome variables. The difference score is taken in the order that the variables are inputted, in this case PrePain – PostPain . The variable in the w argument is the moderator, in this case inflame . When there is only one moderator, the researcher can use either Model=2 or Model=3; the results will not differ. The two additional commands will be explained in the probing section. See the model templates and documentation at https://www.akmontoya.com for more detail and instructions for SAS.

**Fig. 1:**
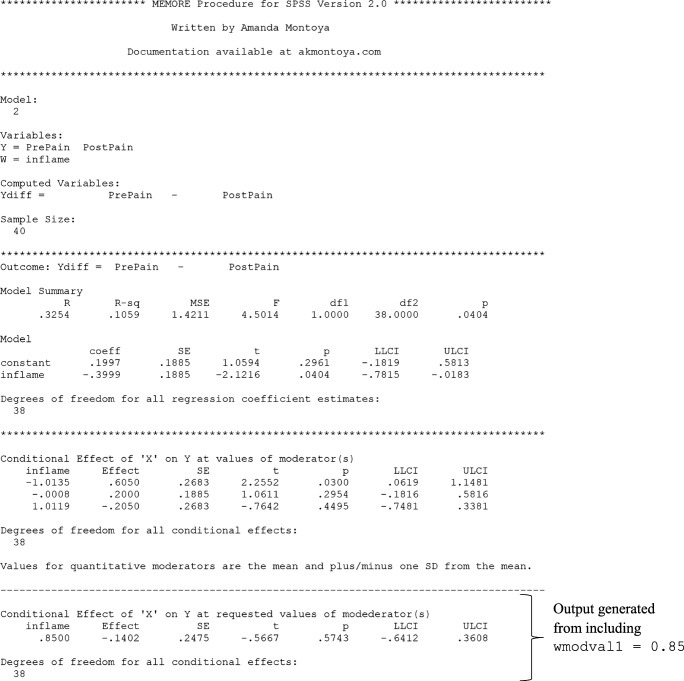

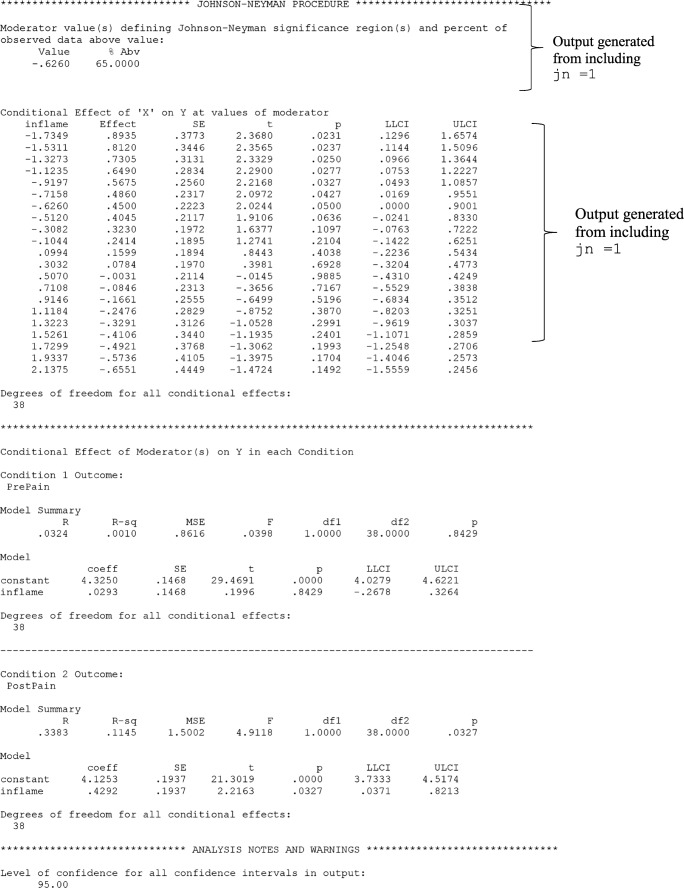
MEMORE SPSS output for simple moderator model generated from theMEMORE command line used for estimation. Output explores a model that allows the effect of treatment on pain (  vs.  ) to be moderated by baseline inflammation ()

See Fig. 1 for the SPSS output. Note that the inflame variable is mean-centered, such that zero is the sample average. The first section of the output has information about the model, variables, calculated variables, and sample size. This section should always be looked over carefully to make sure the intended model is estimated.

The second section of the output includes the results of estimating Eq. 4. The overall model information includes estimated *R*^2^ and a test of the overall variance explained. The second part of this section is a table of all the regression coefficients and their associated significance tests and confidence intervals. Each row corresponds to the results for the predictor denoted in the left column, where “constant” denotes the intercept. The estimated regression equation is

5 $$ {\widehat{Y}}_{pre}-{\widehat{Y}}_{post}={\widehat{Y}}_D=0.20-0.40{W}_i $$

The estimate of the intercept, $\widehat{b_0}=0.20$, means that when the inflammation score is 0 (the sample mean for inflammation), the expected difference in pain is 0.20. Pain is expected be 0.20 units lower after treatment, but this effect is not significantly different from zero, *t*(38) = 1.06, *p* = .30. Additionally, for each unit increase in inflammation, there is a 0.40 unit decrease in the *difference in pain*, *t*(38) = –2.12, *p* = .04*.* The difference was constructed by subtracting post-treatment pain from pre-treatment pain, so lower scores reflect greater post-treatment pain relative to pre-treatment pain. If the treatment is considered effective when post-treatment pain is low relative to pre-treatment pain, then the treatment is less effective for individuals with higher baseline inflammation.

This information alone is very useful, but the researchers may have additional questions. Is the treatment still effective for people with high inflammation, just less so? How much is inflammation related to pre-treatment or post-treatment pain? These questions can be answered by probing the interaction. Next, I discuss how to probe an interaction between a repeated measures factor and between-participant variable.

### Probing the interaction

Just as in between-participant designs, the simple-slopes and Johnson–Neyman procedures can be used to probe moderation effects in two-instance repeated measures designs, though they have not been described in this context before. Two relationships can be probed: the effect of condition on the outcome variable at different values of *W*, and the effect of *W* on the outcome variable in different conditions. I will treat each of these separately, describing both the simple-slopes method and the Johnson–Neyman procedure where they apply.

#### Probing the effect of condition on the outcome

Researchers may be interested in estimating and conducting inference on the effect of condition at specific values of the between-participant variable *W* (e.g., estimating the expected difference, pre-treatment to post-treatment, in pain for an individuals with a specific inflammation score). How well does this treatment work for those who have relatively high inflammation, or for those with relatively low inflammation? A test of interaction examines if the difference in pain differs for those with varying levels of inflammation. However, a test of interaction does not estimate the treatment effect for individuals with a specific score on inflammation. Do people with high inflammation show little difference in pain from a treatment because inflammation reflects a high physical irritation that may not be relieved by behavioral interventions? Do people with low inflammation benefit from the intervention? These questions can be tested by using probing, by estimating the effect of condition at specific values of the between-participant variable using the simple-slopes method. Alternatively, regions of significance can be defined using the Johnson–Neyman method. This analysis would show both where along the between-participant variable any effects of condition on the outcome were significant and where the effects were not statistically significant.

##### Simple slopes

The simple-slopes method relies on choosing a point on the between-participant variable *W*, say *w*, then estimating the effect of condition on the outcome at the specific value *W* = *w*. Based on Eq. 4, the best estimate of the effect of condition on the outcome at a specific value of *W* is ${\widehat{\theta}}_{C\to Y}(W)={\widehat{b}}_0+{\widehat{b}}_3W$, where *C* denotes condition and ${\widehat{\theta}}_{C\to Y}(W)$ denotes the estimated effect of condition on the outcome variable *Y* as a function of *W*. This is the estimate of the difference in the outcome variables between conditions at a specific value of *W*. The variance of ${\widehat{\theta}}_{C\to Y}(W)$ can be estimated as^2^

$$ \widehat{\mathit{\operatorname{var}}}\left({\widehat{\theta}}_{C\to Y}(W)\right)=\widehat{\mathit{\operatorname{var}}}\left({\widehat{b}}_0\right)+{W}^2\widehat{\mathit{\operatorname{var}}}\left({\widehat{b}}_1\right)+2W\widehat{\mathit{\operatorname{cov}}}\left({\widehat{b}}_0,{\widehat{b}}_1\right). $$

The estimated variance of ${\widehat{\theta}}_{C\to Y}(W)$ is a function of the chosen value of the moderator, *W*, the estimated variance of ${\widehat{b}}_0$, $\widehat{\mathit{\operatorname{var}}}\left({\widehat{b}}_0\right)$, the estimated variance of ${\widehat{b}}_1$, $\widehat{\mathit{\operatorname{var}}}\left({\widehat{b}}_1\right)$, and the estimated covariance between ${\widehat{b}}_0$ and ${\widehat{b}}_1$,$\widehat{\mathit{\operatorname{cov}}}\left({\widehat{b}}_0,{\widehat{b}}_1\right)$. The estimates of the variances and covariances of the regression coefficients are available through most statistical packages that estimate regression models. However, typical programs used to conduct regression analysis will not calculate ${\widehat{\theta}}_{C\to Y}(W)$ or $\widehat{\mathit{\operatorname{var}}}\left({\widehat{\theta}}_{C\to Y}(W)\right)$ without additional work by the researcher.

The ratio of the estimate of *θ*_*C* → *Y*_(*W*) to its standard error is *t*-distributed with *n* − *q* − 1 degrees of freedom, where *n* is the number of observations and *q* is the number of predictors in the regression model. In the case of Eq. 4, *q* = 1. Specific values of *W* can be plugged into the equation for ${\widehat{\theta}}_{C\to Y}(W)$ and its $\widehat{\mathit{\operatorname{var}}}\left({\widehat{\theta}}_{C\to Y}(W)\right)$. The ratio $\frac{{\widehat{\theta}}_{C\to Y}(W)}{\sqrt{\widehat{\mathit{\operatorname{var}}}\left({\widehat{\theta}}_{C\to Y}(W)\right)}}$ can be calculated and compared to a critical *t*-statistic with the appropriate degrees of freedom. Alternatively, a *p* value can be calculated from the calculated *t*-statistic.

##### Simple slopes with MEMORE

In the chronic-pain example, probing the effect of instance on the outcome at values of the between-participant moderator means estimating the effect of treatment on pain at different values of baseline inflammation. Suppose the researchers are particularly concerned with knowing if the treatment is still effective for those with high inflammation. They could choose to probe the effect of treatment on pain at the 80th percentile of inflammation, which corresponds to a score of 0.85 on the inflammation measure. Based on the regression results, an estimate of the effect of treatment on pain levels conditional on inflammation can be calculated at 0.85. The estimates of the intercept and regression coefficient for inflammation can be drawn from the regression results in Eq. 5 or Fig. 1.

$$ {\widehat{\theta}}_{C\to Y}(0.85)=0.20-0.40(0.85)=-0.14 $$

This means that individuals who score 0.85 on inflammation are expected to have post-treatment pain levels 0.14 points higher than their pre-treatment pain levels. But is this difference statistically significant? First the variance of the estimate of the conditional effect must be estimated. The variances of each of the regression coefficients are just the squares of their standard errors, which can be extracted from the results in Fig. 1. The covariance between $\widehat{b_0}$ and $\widehat{b_1}$ is exactly zero.

$$ \widehat{\mathit{\operatorname{var}}}\left({\widehat{\theta}}_{C\to Y}(0.85)\right)={0.19}^2+{0.85}^2{0.19}^2+2(0.85)(0)=0.062 $$

$$ \frac{{\widehat{\theta}}_{C\to Y}(0.85)}{\sqrt{\widehat{\mathit{\operatorname{var}}}\left({\widehat{\theta}}_{C\to Y}(0.85)\right)}}=\frac{-0.14}{\sqrt{0.062}}=-0.56 $$

The probability that a *t*-statistic with 38 degrees of freedom is as or more extreme than 0.56 is *p* = .58. All these calculations can be done in MEMORE by including the  argument in the command line. Figure 1 denotes the specific section of the output that corresponds to this command, including a table similar to the one described in the previous section, with estimates of the conditional effect of condition on the outcome at the requested values of the moderator and accompanying information for inference. In addition, MEMORE probes at the mean as well as plus and minus one standard deviation from the mean of the moderator by default (see Fig. 1, “Conditional Effect of ‘X’ on Y at requested values of moderator(s)” heading). See the documentation for additional probing options. The obvious follow-up question after probing at this specific point is then, for what values of inflammation is there a statistically significant effect of treatment on pain?

##### Johnson–Neyman procedure

Just as in between-participant moderation, the ratio of ${\widehat{\theta}}_{C\to Y}(W)$ to its standard error can be used to calculate the point(s) along the range of *W* where the ratio is exactly equal to the critical *t* value. These points mark the boundaries of significance for the relationship between condition and the outcome. By solving for these points, the Johnson–Neyman technique defines the pattern of significance for the relationship between condition and the outcome across the entire range of *W*.

.By setting the absolute value of the ratio of ${\widehat{\theta}}_{C\to Y}(W)$ to its standard error equal to the critical *t* value and solving for *W*, these points can be found using basic algebra. The critical *t* value will be denoted as ${t}_{\frac{\alpha }{2}, df}^{\ast }$. Ratios greater than ${t}_{\frac{\alpha }{2}, df}^{\ast }$ will be significant at level *α*.

$$ {t}_{\frac{\alpha }{2},n-q-1}^{\ast }=\left|\frac{{\widehat{\theta}}_{C\to Y}(W)}{\sqrt{\widehat{\operatorname{var}}\left({\widehat{\theta}}_{C\to Y}(W)\right)}}\right| $$

$$ {t}_{\frac{\alpha }{2},n-q-1}^{\ast }=\left|\frac{{\widehat{b}}_0+{\widehat{b}}_1W}{\sqrt{\widehat{\operatorname{var}}\left({\widehat{b}}_0\right)+{W}^2\widehat{\operatorname{var}}\left({\widehat{b}}_1\right)+2W\widehat{\operatorname{cov}}\left({\widehat{b}}_0,{\widehat{b}}_1\right)}}\right| $$

Squaring both sides eliminates the absolute value sign.

$$ {t}_{\frac{\alpha }{2},n-q-1}^{\ast^2}=\frac{\widehat{\Big({b}_0}+\widehat{b_1}W\Big){}^2\ }{\widehat{\operatorname{var}}\left({\widehat{b}}_0\right)+{W}^2\widehat{\operatorname{var}}\left({\widehat{b}}_1\right)+2W\widehat{\operatorname{cov}}\left({\widehat{b}}_0,{\widehat{b}}_1\right)} $$

Rearrange the terms to get a quadratic form,

$$ 0=\left({b}_0^2-{t}_{\frac{\alpha }{2},n-q-1}^{\ast^2}\widehat{\mathit{\operatorname{var}}}\left({\widehat{b}}_0\right)\right)+\left(2{\widehat{b}}_1{\widehat{b}}_0-2{t}_{\frac{\alpha }{2},n-q-1}^{\ast^2}\widehat{\mathit{\operatorname{cov}}}\left({\widehat{b}}_0,{\widehat{b}}_1\right)\right)W+\left({\widehat{b}}_1^2-{t}_{\frac{\alpha }{2},n-q-1}^{\ast^2}\widehat{\mathit{\operatorname{var}}}\left({\widehat{b}}_1\right)\right){W}^2, $$

and the quadratic formula can be used to show that the solutions to this equation are

$$ {W}_{JNk}=\frac{-\left(2{\widehat{b}}_1{\widehat{b}}_0-2{t}_{\frac{\alpha }{2},n-q-1}^{\ast^2}\widehat{\mathit{\operatorname{cov}}}\left({\widehat{b}}_0,{\widehat{b}}_1\right)\right)\pm \sqrt{{\left(2{\widehat{b}}_1{\widehat{b}}_0-2{t}_{\frac{\alpha }{2},n-q-1}^{\ast^2}\widehat{\mathit{\operatorname{cov}}}\left(\widehat{b_0},{\widehat{b}}_1\right)\right)}^2-4\left({\widehat{b}}_0^2-{t}_{\frac{\alpha }{2},n-q-1}^{\ast^2}\widehat{\mathit{\operatorname{var}}}\left({\widehat{b}}_0\right)\right)\left({\widehat{b}}_1^2-{t}_{\frac{\alpha }{2},n-q-1}^{\ast^2}\widehat{\mathit{\operatorname{var}}}\left({\widehat{b}}_1\right)\right)}}{2\left({\widehat{b}}_1^2-{t}_{\frac{\alpha }{2},n-q-1}^{\ast^2}\widehat{\mathit{\operatorname{var}}}\left({\widehat{b}}_1\right)\right)} $$

In its mathematical form, there are always two solutions to this equation; however, these two solutions are not always interpretable. Just as in the between-participant case, solutions can be imaginary or fall outside of the range of the observed data, neither of which should be interpreted. Even when transition points are found within the range of the data, it is important to note how much of the data is above or below these points, in order to determine how much to trust them. Without data surrounding the Johnson–Neyman points, there is no evidence that the observed trend continues outside the range of the observed data, and thus no evidence that these points are either meaningful or interpretable.

The equation above looks fairly tedious to implement by hand, and computing these values by hand could result in rounding errors. Nonetheless, there is a closed-form solution for these points, and these points of transition can be found using MEMORE.

##### Johnson–Neyman with MEMORE

In the chronic-pain example, it may be useful to find the scores on inflammation such that the treatment is effective at reducing pain, on the basis of a statistically significant difference. MEMORE calculates the Johnson–Neyman points of transition and prints a table of probed values to help researchers understand what ranges of the moderator show significant (and nonsignificant) effects of condition on the outcome. Including the term  in the command line calls for the Johnson–Neyman procedure. Figure 1 shows an example of the output with the chronic-pain data. The critical *t*-statistic for 38 degrees of freedom and *α* = .05 (the default) is 2.02, so the two solutions for the transition points are

$$ J{N}_1=-0.63;J{N}_2=11.79. $$

The second point is well outside the observed range of inflammation (Range = –1.73 to 2.14); however, the first point is within the observed range of the data. The first part of the “Johnson–Neyman Procedure” portion of the MEMORE output includes the Johnson–Neyman points of transition as well as the percentage of the data that fall above that point. MEMORE does not print any Johnson–Neyman solutions that are outside of the observed range of the data. Since one of the solutions, 11.79, was outside the range of the data, MEMORE only printed one Johnson–Neyman solution. The point –0.63 is the transition between the significant and nonsignificant regions. The second part of this portion of the output is a table of probed values that helps give researchers a sense of the pattern of effects across the range of the moderator. The table indicates that points above –0.63 are significant, and those below are nonsignificant.

**Fig. 2:**
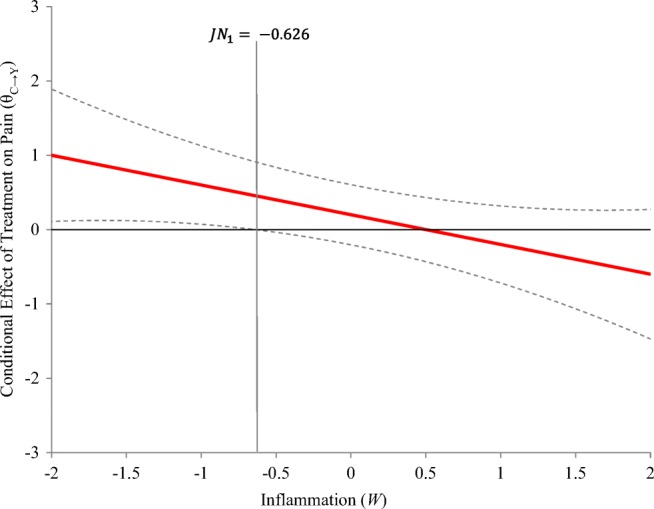
Graph of the conditional effect of treatment (*C*) on pain (*Y*) as a linear function of inflammation (*W*) including the Johnson–Neyman transition point (*JN*). The *JN* point is where the confidence interval around the condition effect intersects zero on the *y*-axis

A helpful way to use the Johnson–Neyman results is to graph the conditional effect of treatment on pain across the range of the moderator, inflammation. If a confidence interval is included around this line, it is easy to tell that the Johnson–Neyman transition points are the points at which the confidence interval around the conditional effect intersects zero on the *y*-axis, marking no significant effect of treatment on condition (see Fig. 2). This kind of visualization can be helpful in understanding an interaction. Because a negative effect of treatment on condition means that post-treatment pain is higher than pre-treatment pain (*Y*_*D*_ < 0), scores below zero indicate an ineffective treatment, or even a treatment that increases pain over time. Positive scores, on the other hand, mean that pain after treatment is lower than pain before treatment. On the basis of Fig. 2, those with inflammation scores below –0.63 are expected to show significant improvement (reduction) in pain after the treatment.

#### Probing the effect of the moderator on the outcome

Estimating the relationship between the between-participant variable *W* and the outcome in each of the different conditions is much simpler than probing the effect of condition on the outcome. In Eqs. 2 and 3, *b*_11_ and *b*_12_ represent the relationship between *W* and *Y* in the first and second conditions, respectively. There is no need to condition on a specific value of a variable and then derive the variance of the conditional estimate. By estimating Eqs. 2 and 3 separately, ${\widehat{b}}_{11}$ and ${\widehat{b}}_{12}$ and their corresponding hypothesis tests are conditional estimates of the relationship between *W* and *Y* and tests of whether these relationship are different from zero. This is equivalent to the simple-slopes method and can conveniently be conducted in any regression program.

Probing the effect of *W* on *Y* is automatically conducted in MEMORE (see Fig. 1, “Conditional Effect of Moderator(s) on Y in each Condition” heading). Consider two individuals who are one unit different from each other on baseline inflammation. The individual with higher baseline inflammation is expected to be 0.0293 units higher in pain levels at baseline. This effect is not significantly different from zero, *t*(38) = 0.20, *p* = .84. However, in Condition 2 (i.e., post-treatment measurement period), a one-unit difference in baseline inflammation is related to a 0.43 unit difference in post-treatment pain levels, where higher baseline inflammation is related to higher post-treatment pain levels. This effect is significantly different from zero, *t*(38) = 2.22, *p* = .03. This is particularly interesting because pre-treatment pain and baseline inflammation were both measured in the same visit. One might expect that pre-treatment inflammation would be more related to pain before treatment than after. However, this shows clear evidence that the relationship is stronger and more positive for post-treatment pain, suggesting that some aspect of the treatment may be less effective for participants with high baseline inflammation. The Johnson–Neyman method cannot be used, because condition is not a continuous variable.

This completes our discussion of probing interactions between a repeated measures factor and a between-participant variable in two-instance repeated measures designs. Next, we move to a discussion of models that incorporate multiple moderators. There I will use additional potential moderators from Lasselin et al. (2016) as an example.

## Multiple-moderator models

Judd et al. (2001, 1996) introduced the regression-based approach to testing interactions between a repeated measures factor and a single between-participant variable. However, they did not generalize beyond a single moderator. When I refer to multiple moderators I mean two or more distinct variables that could impact the relationship between *X* and *Y*, rather than the same moderator measured multiple times. Extensions to multiple moderator models are very important as currently researchers often test a variety of single moderator models separately or conduct subgroup analyses (e.g., Blanco, Barberia, & Matute, 2015; Buunk, Ybema, Van Der Zee, Schaufeli, & Gibbons, 2001). Using separate models or subgroups analysis results in issues of confounding of interaction effects, and does not allow simultaneous testing of multiple moderators and thus can result in imprecise theories about moderation. Testing multiple moderators all together is more parsimonious, and gives the most detailed picture of how the moderators together interact with the focal predictor and each other to predict the outcome. Lasselin et al. (2016) posited that the degree to which effectiveness of treatment depends on inflammation might itself depend on whether the participants received AR or ACT (a three-way interaction), but they did not have the tools available to test this hypothesis. We will test that question in this section.

Multiple-moderator models can be described as two types. The first type is additive moderation, which involves multiple two-way interactions (see Table 1 for a comparison of simple, additive, and multiplicative moderation models). Just as in the simple moderation model (Eq. 4), the researcher needs two observations of the outcome (one in each instance), as well as a single observation on each of the moderators. The moderator variables can be either randomized (e.g., type of therapy) or observed (e.g., baseline inflammation) variables. In additive moderation, the moderators are not allowed to interact with each other. In multiplicative moderation, interactions among the moderators are included. For example, if there were two moderators, all two-way interactions would be included, and the three-way interaction between *X*, *W*_1_, and *W*_2_ would also be included (Hayes, 2018). In this section, I generalize the simple moderation model for two-instance repeated measures designs to models with two moderators, though these methods can be generalized to any number of moderators. MEMORE will estimate and test additive and multiplicative moderation with up to five moderators. I provide an example using the data from Lasselin et al. (2016) with multiple moderators.

**Table 1:**
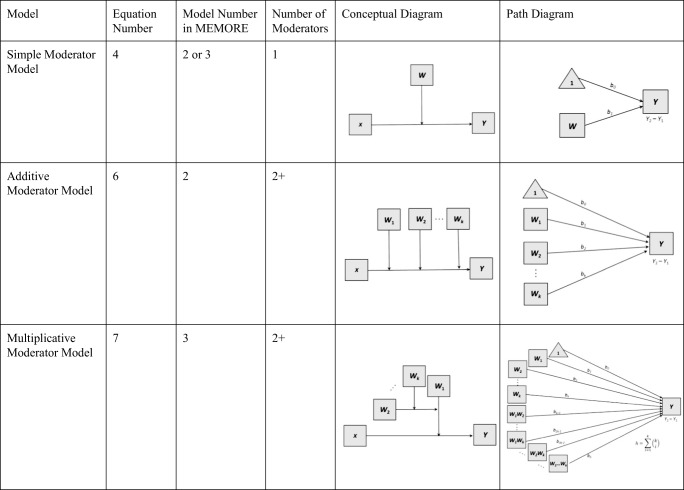
Comparison of three types of moderation models for two-instance repeated measures designs

### Additive moderation

Just as in Judd et al. (2001, 1996), the additive model begins with a model for each outcome in each condition. Additive moderation suggests that the effect of each moderator, *W*_1_ and *W*_2_, depends on the condition, but the effect of each moderator does not depend on the other moderator.

$$ {Y}_{i1}={b}_{01}+{b}_{11}{W}_{1i}+{b}_{21}{W}_{2i}+{\epsilon}_{i1} $$

$$ {Y}_{i2}={b}_{02}+{b}_{12}{W}_{1i}+{b}_{22}{W}_{2i}+{\epsilon}_{i2} $$

The effect of each moderator is allowed to vary by condition, but the effect of each moderator is not a function of the other moderator (i.e., the moderators do not interact with each other). Taking the difference between these equations allows us to test a moderation hypothesis.

6 $$ {\displaystyle \begin{array}{c}{Y}_{i2}-{Y}_{i1}={b}_{02}-{b}_{01}+\left({b}_{12}-{b}_{11}\right){W}_{1i}+\left({b}_{22}-{b}_{21}\right){W}_{2i}+{\epsilon}_{i2}-{\epsilon}_{i1}\\ {}{Y}_{Di}={b}_0+{b}_1{W}_{1i}+{b}_2{W}_{2i}+{\epsilon}_i\end{array}} $$

Equation 6 contains the coefficients of greatest interest *b*_1_ = *b*_12_ − *b*_11_ and *b*_2_ = *b*_22_ − *b*_21_, as these reflect the degree to which condition moderates the effect of each moderator. By symmetry, this is also how each moderator impacts the effect of condition on the outcome. Hypothesis tests on the estimates of *b*_1_ and *b*_2_ will indicate whether the effects of *W*_1_ and *W*_2_ on *Y* differ by condition. Again, the symmetry argument applies: If ${\widehat{b}}_1$ and ${\widehat{b}}_2$ are significantly different from zero, this indicates that the effect of condition depends on *W*_1_ and *W*_2_, respectively. This method can be generalized to any number of moderators.

A particularly useful application of additive moderation is when there is a single conceptual moderator, but it is multicategorical (more than two groups). In this case, the multicategorical moderator can be coded into *k*–1 variables, where *k* is the number of groups, using a coding system such as indicator or Helmert coding. Each of these variables can be included as a separate moderator, and the test of *R*^2^ would be a test of omnibus moderation (Hayes & Montoya, 2017).

Multiple-moderator models can be probed at different sets of values of the moderators or in difference conditions, just as a single moderator model can be probed. In additive moderation, the conditional effect of each moderator remains relatively simple, whereas the conditional effect of condition becomes more complex. For example, in the case of two moderators the conditional effect of *W*_1_ would be

$$ {\theta}_{W_1\to Y}(C)={b}_{1c} $$

and the variance would be $\mathit{\operatorname{var}}\left({\widehat{b}}_{1c}\right)$. Still, the effect of *W*_1_ depends the condition *C*, but it does not depend on the other moderator *W*_2_. The conditional effect of condition would be

$$ {\theta}_{C\to Y}\left({W}_1,{W}_2\right)={b}_0+{b}_1{W}_1+{b}_2{W}_2 $$

The standard errors for these effects become more complex as the number of moderators increase. However, they are easily derived using matrix algebra, and these methods generalize to any number of moderators. Consider the coefficients from the model as a vector $\overset{\rightharpoonup }{b}$, where

$$ \overset{\rightharpoonup }{b}=\left[{b}_0\kern0.5em {b}_1\kern0.5em {b}_2\right]. $$

Additionally, consider the covariance matrix of the parameter estimates to be Σ, where

$$ \Sigma =\left[\begin{array}{ccc}\mathit{\operatorname{var}}\left({\widehat{b}}_0\right)& \mathit{\operatorname{cov}}\left({\widehat{b}}_0,{\widehat{b}}_1\right)& \mathit{\operatorname{cov}}\left({\widehat{b}}_0,{\widehat{b}}_2\right)\\ {}\mathit{\operatorname{cov}}\left({\widehat{b}}_0,{\widehat{b}}_1\right)& \mathit{\operatorname{var}}\left({\widehat{b}}_1\right)& \mathit{\operatorname{cov}}\left({\widehat{b}}_1,{\widehat{b}}_2\right)\\ {}\mathit{\operatorname{cov}}\left({\widehat{b}}_0,{\widehat{b}}_2\right)& \mathit{\operatorname{cov}}\left({\widehat{b}}_1,{\widehat{b}}_2\right)& \mathit{\operatorname{var}}\left({\widehat{b}}_2\right)\end{array}\right] $$

The parameter *θ*_*C* → *Y*_ (*W*_1_, *W*_2_) is a linear combination of the parameters defined by a vector that can be called $\overset{\rightharpoonup }{l}$, where

$$ \overset{\rightharpoonup }{l}=\left[1\kern0.5em {W}_1\kern0.5em {W}_2\right] $$

such that

$$ {\theta}_{C\to Y}\left({W}_1,{W}_2\right)=\overset{\rightharpoonup }{b}\overset{\rightharpoonup }{l^{\prime }}=\left[{b}_0\kern0.5em {b}_1\kern0.5em {b}_2\right]\left[\begin{array}{c}1\\ {}{W}_1\\ {}{W}_2\end{array}\right]={b}_0+{b}_1{W}_1+{b}_2{W}_2 $$

I use the prime symbol to mean “transpose.” The variance of ${\widehat{\theta}}_{C\to Y}\left({W}_1,{W}_2\right)$ is

$$ \mathit{\operatorname{var}}\left({\theta}_{C\to Y}\left({W}_1,{W}_2\right)\right)=\overset{\rightharpoonup }{l^{\prime }}\Sigma \overset{\rightharpoonup }{l} $$

The estimate of the variance of the conditional effect of condition is calculated by using the estimate of Σ. This is a general procedure for finding the standard error of any conditional effect, and only requires that the researcher identify $\overset{\rightharpoonup }{l}$, the contrast vector that identifies the conditional effect of interest. Researchers can use MEMORE to calculate these effects automatically using the , and  arguments (see the documentation at https://www.akmontoya.com).

### Multiplicative moderation

Multiplicative moderation is when the moderators interact with each other as well as the repeated measures factor. This means that the model of *Y* in each condition includes interaction terms, adding complexity to the model. A model is defined for the outcome in each condition:

$$ {Y}_{i1}={b}_{01}+{b}_{11}{W}_{1i}+{b}_{21}{W}_{2i}+{b}_{31}{W}_{1i}{W}_{2i}+{\epsilon}_{i1} $$

$$ {Y}_{i2}={b}_{02}+{b}_{12}{W}_{1i}+{b}_{22}{W}_{2i}+{b}_{32}{W}_{1i}{W}_{2i}+{\epsilon}_{i2} $$

In these models, we can think of the relationship between *W*_1_ and *Y* in each condition as a function of *W*_2_, or the relationship between *W*_2_ and *Y* as a function of *W*_1_. The new terms *b*_31_ and *b*_32_ represent the degree to which *W*_1_ and *W*_2_ interact in their respective conditions. The researcher may be interested in whether the interaction between *W*_1_ and *W*_2_ differs across conditions (i.e., is there a three-way interaction between condition, *W*_1_, and *W*_2_?). This could be tested by examining if *b*_31_ = *b*_32_. To test this hypothesis, the difference between the equations can be used to define coefficients that estimate the parameters of interest.

7 $$ {\displaystyle \begin{array}{l}{Y}_{i2}-{Y}_{i1}={b}_{02}-{b}_{01}+\left({b}_{12}-{b}_{11}\right){W}_{1i}+\left({b}_{22}-{b}_{21}\right){W}_{2i}+\left({b}_{32}-{b}_{31}\right){W}_{1i}{W}_{2i}+{\epsilon}_{i2}-{\epsilon}_{i1}\\ {}{Y}_{Di}={b}_0+{b}_1{W}_{1i}+{b}_2{W}_{2i}+{b}_3{W}_{1i}{W}_{2i}+{\epsilon}_i\end{array}} $$

Estimating Eq. 7 using a linear regression program would yield estimates of each of these coefficients along with inferential statistics. As was mentioned above, a test on ${\widehat{b}}_3$ would be a test of whether there is a three-way interaction between condition, *W*_1_, and *W*_2_. As additional moderators are added, the same method could be used to test higher order interactions that include a repeated measures factor.

In all previous analyses, tests on *b*_1_ and *b*_2_ were tests of two-way interactions. Now they are tests of *conditional two-way interactions*. The coefficient *b*_11_ is the effect of *W*_1_ on *Y*_1_ conditional on *W*_2_ being zero, and *b*_12_ is the effect of *W*_1_ on *Y*_2_ conditional on *W*_2_ being zero. The difference between *b*_11_ and *b*_12_, *b*_1_, is the degree to which the conditional effect of *W*_1_ on *Y* conditional on *W*_2_ being zero differs across conditions. Because the degree to which *W*_1_ affects *Y* in any given condition is allowed to depend on *W*_2_, there is no single effect of *W*_1_ on *Y* in a specific condition. So, *b*_1_ reflects a conditional two-way interaction (between condition and *W*_1_) conditional on *W*_2_ being zero. Similarly, *b*_2_ reflects the degree to which the effect of *W*_2_ on *Y* conditional on *W*_1_ being zero differs across conditions.

I’ve described how to test a three-way interaction between a repeated measures factor and two between-participant variables. This method can be generalized to any number of moderators. In addition to the test of interaction, probing can be used to better understand the pattern of effects. Especially with higher order interactions, understanding the pattern of effects throughout the range of the moderators can be very difficult by just examining the coefficients. Both Johnson–Neyman and simple-slopes probing methods can be generalized to higher order interactions, though the simple-slopes approach is often more interpretable as the researcher can choose specific sets of values for the moderators and estimate the effect of condition on the outcome. Generalizations of the Johnson–Neyman procedure to multiple-moderator models involve either higher dimensional spaces (Hunka & Leighton, 1997) or regions of significance for interactions (Hayes, 2018), both of which can be very difficult to interpret. Because of this I focus on using the pick-a-point procedure in multiple moderator models.

When moderation is multiplicative, probing becomes even more important because the effect of each moderator will depend on the value of the other moderators. For example, in the case of two moderators the conditional effect of *W*_1_ would be

$$ {\theta}_{W_1\to Y}\left(C,{W}_2\right)={b}_{1c}+{b}_{3c}{W}_2 $$

and the variance would be

$$ \mathit{\operatorname{var}}\Big({\theta}_{W_1\to Y}\left(C,{W}_2\right)=\mathit{\operatorname{var}}\left({b}_{1c}\right)+{W}_2^2\mathit{\operatorname{var}}\left({b}_{3c}\right)+2{W}_2\mathit{\operatorname{cov}}\left({b}_{1c},{b}_{3c}\right). $$

Now the effect of *W*_1_ is conditional on both the condition *C* and the other moderator *W*_2_. Additionally, the conditional effect of condition would be

$$ {\theta}_{C\to Y}\left({W}_1,{W}_2\right)={b}_0+{b}_1{W}_1+{b}_2{W}_2+{b}_3{W}_1{W}_2. $$

Using the methods outlined in the previous section, we can identify that $\overset{\rightharpoonup }{l}$ is

$$ \overset{\rightharpoonup }{l}=\left[1\kern0.5em {W}_1\kern0.5em {W}_2\kern0.5em {W}_1{W}_2\right] $$

such that

$$ {\theta}_{C\to Y}\left({W}_1,{W}_2\right)=\overset{\rightharpoonup }{b}\overset{\rightharpoonup }{l^{\prime }}=\left[{b}_0\kern0.5em {b}_1\kern0.5em {b}_2\kern0.5em {b}_3\right]\left[\begin{array}{c}1\\ {}{W}_1\\ {}{W}_2\\ {}{W}_1{W}_2\end{array}\right]={b}_0+{b}_1{W}_1+{b}_2{W}_2+{b}_3{W}_1{W}_2 $$

This also means that

$$ \mathit{\operatorname{var}}\left({\widehat{\theta}}_{C\to Y}\left({W}_1,{W}_2\right)\right)=\overset{\rightharpoonup }{l^{\prime }}\Sigma \overset{\rightharpoonup }{l} $$

where Σ is

$$ \Sigma =\left[\begin{array}{cccc}\mathit{\operatorname{var}}\left({\widehat{b}}_0\right)& \mathit{\operatorname{cov}}\left({\widehat{b}}_0,{\widehat{b}}_1\right)& \mathit{\operatorname{cov}}\left({\widehat{b}}_0,{\widehat{b}}_2\right)& \mathit{\operatorname{cov}}\left({\widehat{b}}_0,{\widehat{b}}_3\right)\\ {}\mathit{\operatorname{cov}}\left({\widehat{b}}_0,{\widehat{b}}_1\right)& \mathit{\operatorname{var}}\left({\widehat{b}}_1\right)& \mathit{\operatorname{cov}}\left({\widehat{b}}_1,{\widehat{b}}_2\right)& \mathit{\operatorname{cov}}\left({\widehat{b}}_1,{\widehat{b}}_3\right)\\ {}\mathit{\operatorname{cov}}\left({\widehat{b}}_0,{\widehat{b}}_2\right)& \mathit{\operatorname{cov}}\left({\widehat{b}}_1,{\widehat{b}}_2\right)& \mathit{\operatorname{var}}\left(\widehat{b_2}\right)& \mathit{\operatorname{cov}}\left({\widehat{b}}_2,{\widehat{b}}_3\right)\\ {}\mathit{\operatorname{cov}}\left({\widehat{b}}_0,{\widehat{b}}_3\right)& \mathit{\operatorname{cov}}\left({\widehat{b}}_1,{\widehat{b}}_3\right)& \mathit{\operatorname{cov}}\left({\widehat{b}}_2,{\widehat{b}}_3\right)& \mathit{\operatorname{var}}\left({\widehat{b}}_3\right)\end{array}\right] $$

These calculations are done in MEMORE using the  , and  arguments (see the documentation at https://www.akmontoya.com).

### Example of additive moderation with MEMORE

Lasselin et al. (2016) expressed concerns that perhaps the effects were stronger for participants who went through ACT rather than AR therapy, as had been found in Kemani et al. (2015). By adding in *type of therapy* as a moderator, this hypothesis can be tested. In this analysis, I will use additive moderation. The MEMORE command for this analysis in SPSS would be

**Fig. 3:**
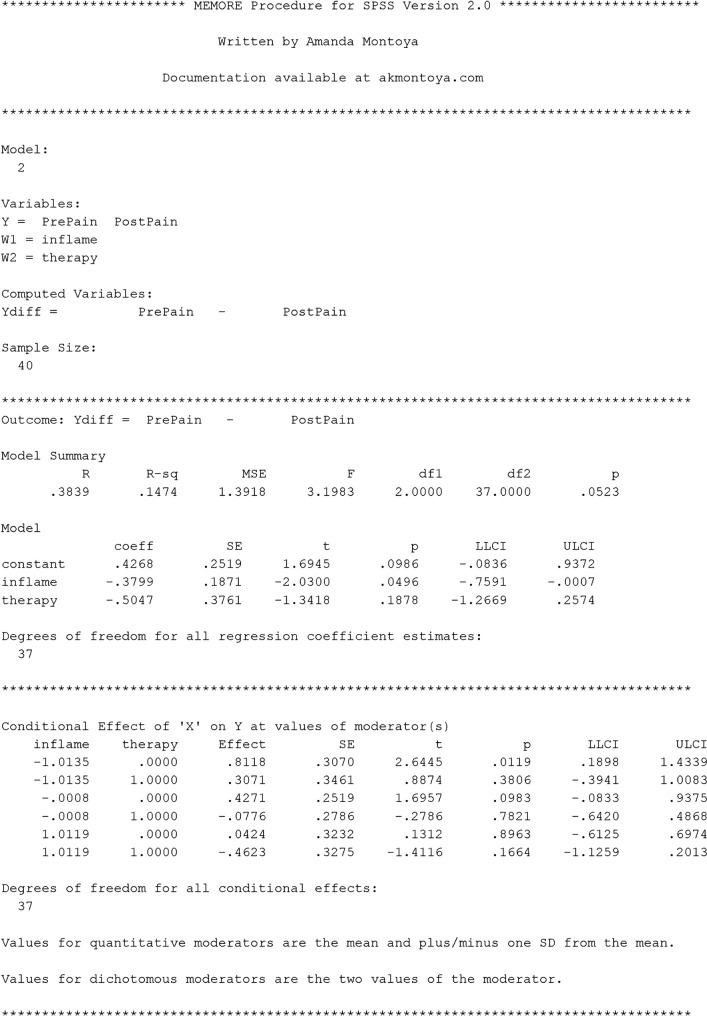

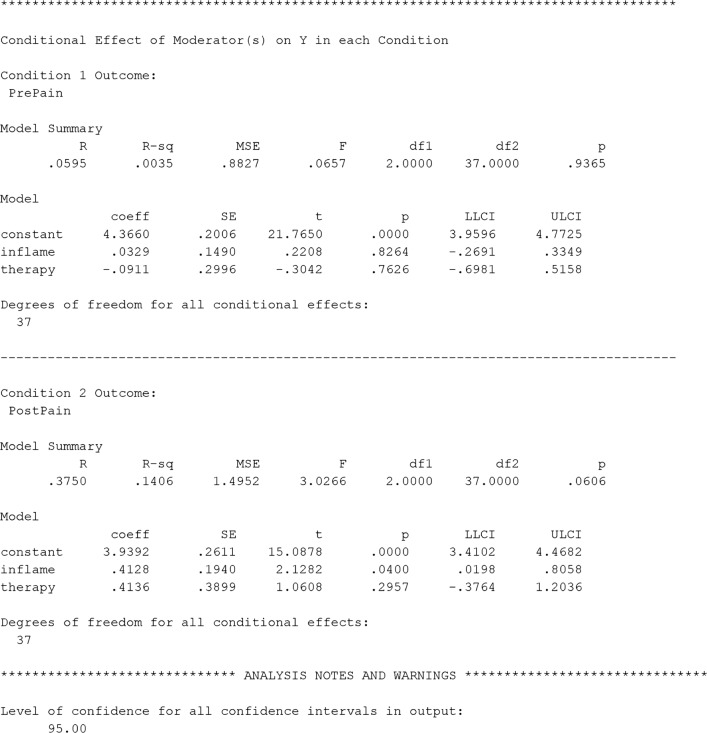
MEMORE SPSS output for an additive moderator model generated from the command line in the text. The output explores a model that allows the effect of treatment on pain (PrePain v+s. PostPain) to be moderated by type of treatment (therapy) and baseline inflammation (inflame)

The additional moderator  is included by adding it to the w list in the MEMORE command, and additive moderation is indicated by using Model 2 (see the documentation for details and SAS commands). Figure 3 contains the output for the command specified above. The  variable is coded so that ACT is 0 and AR is 1. The first section of the output gives information about the model: which variables are assigned to which role, the order of subtraction for the outcome variable, and the sample size.

The second section of the output is the results from estimating Eq. 6 with the data from Lasselin et al. (2016). This table is just like the table from the single-moderator analysis, but now it has multiple predictors. The estimated regression equation is

8 $$ {\widehat{Y}}_D=0.43-0.38{W}_1-0.50{W}_2 $$

where inflammation is *W*_1_ and therapy type is *W*_2_. Pain after treatment is expected to be 0.43 units lower than pain before treatment for those who are average on inflammation (*W*_1_ = 0) and in the ACT condition (*W*_2_ = 0), but this effect is not significant, *t*(37) = 1.69, *p* = .10. As inflammation increases by one unit, the difference between pain before and after treatment decreases by 0.38 units (i.e., treatment becomes less effective), and this effect is just significant, *t*(37) = – 2.03, *p* = .05. Finally, it seems that participants in the AR condition have 0.50 units less difference on pain from pre- to post-treatment, but this effect is not significant, *t*(37) = – 1.34, *p* = .19. If this effect were significant, it would indicate that AR therapy was less effective than ACT therapy at reducing pain, as was suggested by Kemani et al. (2015).

For both the additive and multiplicative moderation models, MEMORE probes the effect of condition at a variety of sets of values of the moderators (see Fig. 3, “Conditional effect of ‘X’ on Y at values of moderator(s)” heading). These results show that the treatment is most effective when inflammation is low (*W*_1_ =  − 1.01) and with ACT therapy (*W*_2_ = 0). For this group, pain levels are expected to decrease 0.81 units over the course of treatment, *t*(37) = 2.64, *p* = .01. However, there is no significant reduction in pain when inflammation is high or with AR therapy.

In this section, I’ve described how to estimate and test moderation in two-instance repeated measures designs with multiple between-participant moderators. Although throughout the article I’ve described methods for testing these models using regression, there are alternative statistical approaches to answering these types of questions. I now turn to some short descriptions of these alternatives and where to learn more about them.

## Alternatives

The methods in this article proposed for testing moderation are not the only possible methods for testing a moderation hypothesis in a two-instance repeated measures design. Two particularly important methods require mention: structural equation modeling and multilevel modeling. Judd et al. (1996) directly compared the regression methods for testing an interaction described in this article to structural equation modeling. Multilevel modeling requires additional multiple observations of each person in each condition, but if this type of data is available, then methods for testing and probing interactions in multilevel models have been discussed in depth in other articles (Bauer & Curran, 2005; Preacher et al., 2006) and could be used.

### Structural equation modeling

**Fig. 4:**
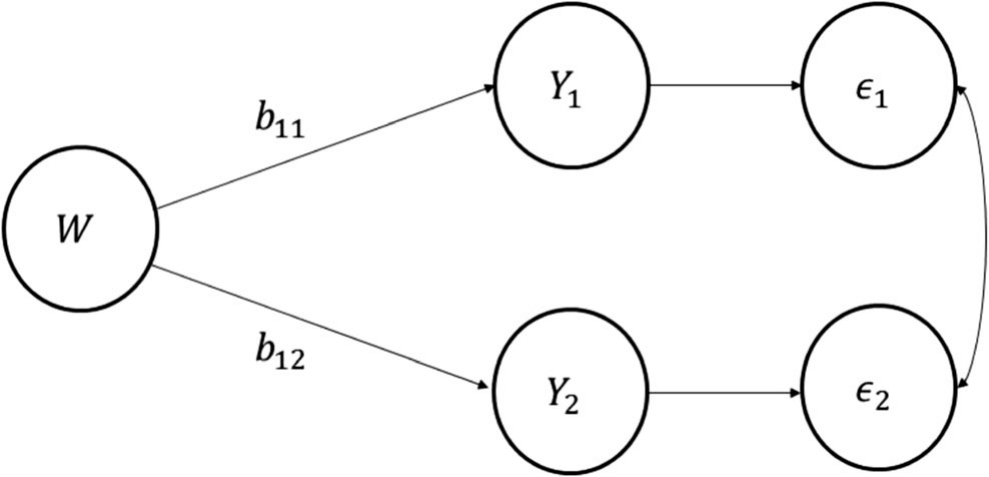
Path diagram representing structural equation model for testing moderation in a two-instance repeated measures design

Judd et al. (1996) compared the approach described in this article to a very basic structural equation modeling (SEM) approach in which the moderator is allowed to predict each of the outcomes, and the residuals in these models are allowed to covary (see Fig. 4). Note the correspondence between Fig. 4 and Eqs. 2 and 3. In a SEM approach, the researcher would estimate the model in Fig. 4, then fix the two paths *b*_11_ and *b*_12_ to be equal, and then use a model comparison approach to test whether the model with free paths fits significantly better than the model with fixed paths. In a SEM approach, this would be done by using a *χ*^2^ goodness-of-fit statistic to compare the two models. Note, though, that the null hypothesis in this structural equation model is the same as in the methods proposed in this article. The concern is whether a model in which *b*_11_ = *b*_12_ describes the data sufficiently, or would it be better to allow the relationship between *W* and *Y* to vary by condition, thus allowing *b*_11_ ≠ *b*_12_.

The SEM analyses could be conducted in a variety of structural equation modeling programs like LISREL, Mplus, AMOS, EQS, and so forth. Some of these programs only use the variance–covariance matrix of the variables to estimate the paths involved. Judd et al. (1996) found that little to no difference in statistical power or Type I errors between the methods proposed in this article and using a SEM approach.

To probe the interactions, the intercept is needed, so the means of the variables are also needed. Many programs have the functionality to accept either the raw data or the mean vector of all the variables in the dataset. Either way, this additional information would be needed to probe the interaction. Mplus allows the researcher to define additional parameters that can be combinations of existing parameters, and will then include inferential tests on these new parameters in the output. By choosing specific values of the moderator to probe at, and defining additional parameters using these values, the simple-slopes method is easy to implement in Mplus. The Johnson–Neyman procedure is not implemented in any of the existing structural equation modeling programs, so this method of probing would not be available in a SEM approach. Below is an example of Mplus code to estimate all the parameters of interest including the conditional effect of therapy on pain at an inflammation level of 0.85. Though there is not a way to get the exact Johnson–Neyman transition points in Mplus, there is a fairly simple way to get plots that align with the Johnson–Neyman procedure. I’ve also included code that creates the Johnson–Neyman plots (similar to the one in Fig. 2). The parameter estimates are identical, but the standard errors are slightly different, because Mplus uses asymptotic variance estimates (denominator of *N*) and ordinary least squares uses sample variance estimates (denominator of *N*–1).

One advantage of SEM is the superior methods available for dealing with missing data. Particularly with the methods described in this article, the use of the difference score means that if individuals have missing data on either observation of the outcome, they will not be included in the analysis when regression analysis is used. SEM allows for methods like full-information maximum likelihood, which can handle missing data without using any imputation methods. This means that individuals with some data can still contribute to the overall estimates.

An additional advantage of SEM approaches is the ability to include latent variables. Often the outcome variable or the moderator is not just one variable, but the combination of many. For example, Lasselin et al. (2016) conducted principal components analysis to create the measure of inflammation. This could have been integrated into the complete analysis by creating a latent variable that was indicated by each of observed inflammation variables, in which case the whole structural equation model, including a latent variable for inflammation, could have been estimated simultaneously. In the case in which the moderator is a latent variable and the only predictor in the model, using a structural equation modeling approach will likely result in better power and more accurate standard errors for coefficient estimates, since regression methods assume that there is no measurement error in the predictor variables. This is not necessarily the case with additive moderation, as measurement error in multiple variables can have varying effects on estimation. In the case of multiplicative moderation, latent moderators will result in latent interactions, an area of research that is still in development (e.g., Cham, West, Ma, & Aiken, 2012; Marsh, Wen, Hau, & Nagengast, 2013).

### Multilevel modeling

Multilevel modeling approaches to two-instance repeated measures designs are only possible if there are multiple replicates per condition. For example, in many cognitive psychology studies, participants see a variety of visual cues that are from two different conditions. When there are many trials per condition, there are many observations of each participant in each condition. With only one observation per participant per condition, the multilevel models will not have enough degrees of freedom to estimate the parameters of interest. The basic premise of the multilevel model, however, is quite similar to those models that were described in this article. The multilevel model used to assess interaction in a two-condition within-participant design would be

$$ {Y}_{ij}={b}_{0i}+{b}_{1i}{X}_{ij}+{\epsilon}_{ij} $$

where

$$ {b}_{0i}={\gamma}_{00}+{\gamma}_{01}{W}_i+{u}_{0i} $$

$$ {b}_{1i}={\gamma}_{01}+{\gamma}_{11}{W}_i+{u}_{1i} $$

Here, *i* denotes individual and *j* indexes repeated measurements of that same individual. *X*_*ij*_ denotes the condition for person *i* during replicate *j*. The coefficients with subscripts *i* are random by person. This is one of the great advantages of multilevel models, in that by including random coefficients, the dependencies among observations from the same person can be taken into account. The equations above may look very different from those used throughout this article, however when they are combined the resulting equation is quite similar to the equation used throughout this article, but also including person-specific errors.

$$ {Y}_{ij}=\left({\gamma}_{00}+{\gamma}_{01}{W}_i+{u}_{0i}\right)+\left({\gamma}_{01}+{\gamma}_{11}{W}_i+{u}_{1i}\right){X}_{ij}+{\epsilon}_{ij} $$

The model above would be quite unstable with only two observations per person. A more stable model would be to fix the random coefficient for *X*_*ij*_; however, this would then not allow for *W* to moderate the effect of *X* on *Y*. But if there are many replicates per person in each condition, these models should prove to be superior to the methods proposed in this article. Bauer and Curran (2005) and Preacher, Curran, and Bauer (2006) provide excellent introductions to moderation analysis in multilevel models and computational tools for both testing and probing interactions using simple-slopes and Johnson–Neyman procedure.

## Extensions

There are a variety of extensions for the methods proposed in this article, which could be useful throughout experimental psychology and other scientific fields. In this section I address a few of the ones that I expect to be of particular interest. Some extensions are described below, but others could provide potentially fruitful future directions of research.

### When *W* is expected to change across instances

Throughout this article I have addressed how to conduct moderation analysis when the moderator is a between-participant variable (measured once); however, researchers may wonder what to do if they believe that their moderator changes across instance. In this case, the researcher might measure the moderator twice, one in each instance. The original article by Judd et al. (1996) addressed “moderation” in this case. Looking more closely, however, the authors revised their approach to testing moderation when the moderator is measured repeatedly, and in their 2001 article they discussed this analysis as *mediation*. The hypothesis of moderation implies that the moderator impacts the relationship between instance and the outcome. This means the moderator should have temporal precedence over instance, and instance should not affect the moderator. If the moderator varies across instances, that means that instance is affecting the moderator. In this case it is difficult to discuss how the moderator affects the relationship between instance and the outcome, when it is clear that instance is affecting the moderator. Judd et al. (2001) addressed how to assess mediation when the mediator is measured in each instance, and Montoya and Hayes (2017) update this approach to the more modern path-analytic approach, providing a computational tool, MEMORE (Model 1), for conducting inference on the indirect effect in these cases.

### Including covariates

In the analysis described in this article, it is unclear how one should include additional covariates or even if those covariates *should* be included. An important aspect of this analysis is that it is a within-person analysis. If there is an effect of a covariate on the outcome and it does not vary across conditions, this covariate will cancel out when taking the difference score. Lasselin et al. (2016) were also interested in how age might impact pain levels. In the equations below I denote Age as *A*. Consider new versions of Eqs. 2 and 3, which now include age as a covariate:

$$ {Y}_{i1}={b}_{01}+{b}_{11}{W}_i+{b}_{21}{A}_i+{\epsilon}_{i1} $$

$$ {Y}_{i2}={b}_{02}+{b}_{12}{W}_i+{b}_{22}{A}_i+{\epsilon}_{i2} $$

In each equation, age is controlled for. However, if the relationship between age and the outcome is the same across conditions (i.e., *b*_21_ = *b*_22_), then when the difference score is taken age will cancel out and is not required in the final model.

$$ {Y}_{i2}-{Y}_{i1}={b}_{02}-{b}_{01}+\left({b}_{12}-{b}_{11}\right){W}_i+\left({b}_{22}-{b}_{21}\right){A}_i+\left({\epsilon}_{i2}-{\epsilon}_{i1}\right) $$

$$ {Y}_{Di}={b}_0+{b}_1{W}_i+{b}_2A+{\epsilon}_i $$

Therefore, if researchers are concerned about controlling for an additional variable, but they do not believe that the effect of that variable depends on condition, then that variable is not needed. If instead they believe that the effect of that variable depends on condition and they want to control for it, then age or any other covariate of interest should be treated as an additional moderator.

### More than two instances

This article has focused on two-instance repeated measures designs; however, there may be situations when there are more than two conditions. Hayes and Montoya (2017) describe how to test moderation and probe moderation in a between-participant design when the focal predictor is multicategorical. In the within-participant case, including additional conditions involves taking contrasts of the conditions rather than difference scores (Judd et al., 2001). Using a structural equation modeling approach, contrasts of interest can be defined and a likelihood ratio test can be used to test the significance of the effect. Once these contrasts are defined, probing the conditional effects is a simple generalization of the work presented in this article; however, no published research has addressed this concern, nor are there computational tools to do so. When there are more than two conditions, there is also the opportunity to probe the omnibus test of group differences, an issue that is still unresolved in the within-participant case.

### Alternative models of change

The analytical approach described in this article relies on difference scores to describe change for each individual. Difference scores can be useful in modeling change; however, they can be insensitive to phenomena like regression toward the mean or ceiling and floor effects (Campbell & Kenny, 1999; Cronbach & Furby, 1970; Jamieson, 1995). Many researchers have suggested abandoning the use of difference scores in favor of alternative methods (e.g., Bonate, 2000; Cronbach & Furby, 1970; Lord, 1963; Twisk & Proper, 2004; but see Rogosa, 1995; Thomas & Zumbo, 2012; Zumbo, 1999). Some alternative models include those based on residualized change scores, in which the second measurement is regressed on the first, and the residuals from this model are then regressed onto the predictors of interest. This method could also be used to test and probe moderation, where instead of predicting the difference score, the residualized change score would be predicted by the moderator.

$$ {Y}_{2i}={i}_{Y_2}+b{Y}_{1i}+{u}_{Y_{2i}} $$

$$ {\widehat{u}}_{Y_{2i}}={b}_0+{b}_1{W}_i+{e}_{Y_{2i}} $$

This model of change corrects for the initial measurement and expected regression toward the mean (Campbell & Kenny, 1999). Another alternative is an autoregressive model (also known as ANCOVA), where the second measure is predicted by the first as well as by other predictors.

$$ {Y}_{2i}={b}_0+{b}_1{Y}_{1i}+{b}_2{W}_i+{e}_{Y_{2i}} $$

It is worth noting that the autoregressive model is equivalent to the difference score model when *b*_1_ = 1 (Brogan & Kutner, 1980). Each of these models represents a different model of change, and it is likely that different methods will perform better or worse depending on what the true model of change is (if there is such a thing). Indeed much of the simulation work in this area has found that the performance of these different models depends on the generating model and no method works optimally for all types of data (Jamieson, 1995; Kisbu-Sakarya, MacKinnon, & Aiken, 2013; Petscher & Schatschneider, 2011). Much of the simulation work has focused on study designs in which individuals are randomly assigned to one of two conditions and measured twice (before and after treatment). The statistical methods described in this article could be used for that type of study, but also for studies involving continuous moderators. Future research could examine how these different models of change perform in investigating questions of moderation in which the moderator is continuous and not randomly assigned.

### Moderated mediation

The integration of mediation and moderation in between-participant designs has become a flourishing topic over the past decade (Edwards & Lambert, 2007; Fairchild & MacKinnon, 2009; Hayes, 2015; Preacher, Rucker, & Hayes, 2007). Perhaps one of the most promising future directions from this research would be the integration of mediation and moderation in two-instance repeated measures designs. Recent work has proposed path-analytic approaches for estimating and testing indirect effects in two-instance repeated measures designs (MacKinnon, 2008; Montoya & Hayes, 2017; Valente & MacKinnon, 2017). By combining methods for mediation and moderation, we can estimate and test the moderation of mechanisms in two-instance repeated measures designs and probe the indirect effects using methods similar to those proposed in this article.

## Conclusion

Previous research on moderation in between-participant designs has established both how to test for interactions/moderation and how to probe these moderation effects to better understand the pattern of effects across the moderator. Easy-to-use computational tools are available as additions to popular statistical software (e.g., SPSS and SAS) or available online and have made the adoption of these methods widespread throughout psychology and other academic research fields. Previous research on moderation analysis in two-instance repeated measures designs had only established how to test for an interaction, and been limited to a single moderator. This article generalized the analysis to include two probing methods, simple-slopes and the Johnson–Neyman procedure, as well as generalized the models to any number of moderators. MEMORE (available at https://www.akmontoya.com) can conduct inferential tests for moderation, probe using both methods described in this article, and test models with up to five moderators, which may either be additive or multiplicative. With these additional innovations and tools for two-instance repeated measures moderation analysis, researchers can now conduct their analyses more thoroughly and accurately, with better understanding of the nature of the interactions they are investigating.

## Footnotes

1 The residuals in this equation (*ϵ*_*i*2_ − *ϵ*_*i*1_) are still normally distributed with constant variance. Assume that the correlation between *ϵ*_*i*1_ and *ϵ*_*j*2_ is *ρ* for all *i* = *j*, and zero for all *i* ≠ *j*; then, ${\epsilon}_{i2}-{\epsilon}_{i1}\sim N\left(0,{\sigma}_1^2+{\sigma}_2^2-2\rho {\sigma}_1{\sigma}_2\right)$.

2 Under the assumptions of linear regression, ${\widehat{b}}_0$ and ${\widehat{b}}_1$ are normally distributed. If *X* and *Y* are normally distributed, *aX* + *bY* is normally distributed, with variance *a*^2^ var(*X*) + *b*^2^ var(*Y*) + 2*ab* cov(*X*, *Y*).
